# Dietary Selenium-Enriched Aquatic Products for Human Health

**DOI:** 10.3390/nu17233640

**Published:** 2025-11-21

**Authors:** Huilong Qiu, Hai Ren, Weijian Wang, Jiaqiang Huang, Lianshun Wang, Xiaomin Jin

**Affiliations:** 1 College of Marine Resources & Environment, Hebei Normal University of Science & Technology, Qinhuangdao 066600, China; qiucau2580@163.com (H.Q.); hairen1982@163.com (H.R.); 2 Institute of Industry-Urban Integration, Qingdao University of Science and Technology, Qingdao 266100, China; 18870768184@163.com; 3 Department of Nutrition and Health, China Agricultural University, Beijing 100193, China; jqhuang@cau.edu.cn; 4 School of Fisheries and Life Science, Dalian Ocean University, Dalian 116023, China

**Keywords:** selenoproteins, aquaculture animals, human health, dietary recommendations

## Abstract

Aquatic products are among the best sources of high-quality protein for humans. Selenium is also an essential trace element for animal growth and development. In animals, selenium primarily exists as selenoproteins, which perform vital physiological functions such as providing antioxidant protection, regulating the immune system, and facilitating growth and reproduction. Twenty-four of the selenoproteins identified in humans have also been found in aquatic animals. As living standards rise and health awareness grows, consumers are increasingly favoring selenium-enriched aquatic products. This article systematically reviews the advantages of these products, selenium levels in different farmed animals, and dietary recommendations. It also explores the functions of selenoproteins in these products. The article emphasizes the importance of selenium for human health and discusses its relationship with health. It also explores the application prospects of selenium-enriched aquatic products. The goal is to provide a scientific basis for utilizing these products and to guide consumers in supplementing their selenium intake.

## 1. Introduction

Selenium is one of the essential trace elements for humans. Within the body, it promotes the synthesis of selenoproteins through genetic control, facilitating the insertion of selenocysteine (Sec), the Selenocysteine Insertion Sequence (SECIS) promotes translation of the codon UGA encoding Sec into selenoproteins, and, to date, 25 selenoproteins have been identified in humans [1,2]. Selenium is crucial for human health, GPX and TXNRD have exhibiting antioxidant properties and enhancing immunity, eliminate reactive oxygen species (ROS) and peroxides; DIO primarily converts thyroxine (T4) secreted by the thyroid gland into the more biologically active triiodothyronine (T3), thereby regulating thyroid hormone levels. Furthermore, selenium prevents cardiovascular diseases, improves reproductive performance and provides anticancer and anti-inflammatory effects [3,4,5,6]. Chronic selenium deficiency can lead to impaired immune regulation, Keshan disease, thyroid dysfunction, reproductive abnormalities, cognitive decline and accelerated ageing [7,8]. Individuals residing in selenium-deficient regions, with limited dietary diversity or in specific physiological or disease states, are particularly susceptible to deficiency [9]. Food is the main source of selenium intake for humans, and maintaining adequate selenium homeostasis is essential for physiological function [10]. Optimizing human selenium intake to prevent selenium deficiency-related diseases is an urgent issue in modern global healthcare. Consequently, the development of selenium-enriched aquatic products has received significant attention both domestically and internationally.

We must adopt a holistic approach to food, sourcing it not only from land, but increasingly from the oceans too. Fisheries are a vital part of this approach, with the oceans acting as a “blue granary”. The development of the fishery industry must continue to advance industrial restructuring, guided by public demand, to provide high-quality, green fishery products [11]. Aquaculture is increasing the global supply of food and has great potential to address malnutrition and diet-related diseases [12,13]. Including more selenium-rich aquatic products in the human diet can help to alleviate selenium deficiency [14,15]. Aquatic products such as fish are also rich in protein, amino acids, vitamins, and unsaturated fatty acids, including eicosapentaenoic acid (EPA) and docosahexaenoic acid (DHA) [16], which play a vital role in brain and eye development. In recent years, research into the biological functions and mechanisms of selenium in aquaculture animals has intensified. Supplementing selenium in feed effectively improves animal growth performance, enhances immunity, and boosts antioxidant and stress-resistance capabilities. The development and application of selenium-enriched functional feeds have established production technologies for selenium-enriched aquaculture animals and pioneered new farming models enriched with selenium, all of which have yielded positive outcomes [14,17,18]. Due to selenium’s vital role in human health, selenium-rich aquatic products are becoming increasingly popular among consumers for their nutritional value and the health benefits associated with selenium.

This paper first compares the selenium content of aquatic products with that of other foods. It then lists the selenium levels in various farmed animal tissues and recommends scientific dietary intake levels for selenium-enriched aquatic products. Finally, it emphasizes the positive role of selenoproteins in aquatic products for human nutrition and health. Finally, it discusses the prospects for developing selenium-enriched aquatic products. The article recommends incorporating these products into daily diets to further enhance consumer awareness of the nutritional and health benefits of selenium in aquatic products.

## 2. Introduction to Selenium-Enriched Aquatic Products

### 2.1. Key Advantages

Selenium-enriched aquatic products are high-quality foods by nature. Fish muscle is rich in high-quality protein and contains all the essential amino acids required by the human body, making it a highly nutritious food. Compared to terrestrial meat proteins, fish protein is more easily digested and absorbed [19]. Fish muscle is also abundant in lipids and polyunsaturated fatty acids (PUFAs), such as omega-3, primarily EPA and DHA, which are beneficial for brain development and cardiovascular health [20]. These components help to prevent and treat cardiovascular disease, boost immunity, and combat inflammation [21]. Furthermore, fish muscle contains various vitamins (folate, vitamins A, D, B2, and B12) and minerals (zinc, potassium, calcium, phosphorus, iodine, iron, and selenium), which offer significant health benefits, including protection against night blindness, anemia, osteoporosis, and maintaining thyroid function [22,23]. Research indicates that the dark meat of yellowfin tuna is an excellent source of selenium from animal products. Its protein fraction exhibits the highest selenium-binding capacity at 15.76 ± 0.04 mg/kg, surpassing the polysaccharide and nucleic acid fractions [24]. Additionally, the organic form of selenium found in aquatic animals is primarily selenomethionine (SeMet), which is bioavailable. This form can participate directly in human protein synthesis and contribute indirectly to physiological processes such as antioxidant defense, immune system function, and thyroid metabolism [25]. In contrast, certain forms of selenium in plants, such as inorganic selenium, selenosugar, and selenopolysaccharide, require hepatic metabolism to convert them into SeMet or Sec before they become bioavailable. Furthermore, the content and chemical forms of selenium in plants can vary significantly across species and their specific metabolic pathways [26,27,28]. The organic selenium forms in selenium-enriched aquatic products can be directly absorbed and transformed, making them more suitable for populations with selenium deficiencies or requiring supplementation. Crucially, the products offer the dual benefits of high-quality protein and selenium nutrition. Compared to selenium-enriched foods such as grains, fruits, vegetables, dairy products, and meats [29], aquatic products (fish, sea cucumbers, and shrimp) have a higher selenium content and can fully meet consumers’ daily requirements for a healthy diet (Table 1). Incorporating selenium-enriched aquatic products into the daily diet allows individuals to obtain high-quality protein nutrition and effortlessly supplement selenium, eliminating the need for additional supplements and providing a convenient method of dietary selenium supplementation.

### 2.2. Selenium-Enriched Aquaculture Animals and Their Selenium Content

As living standards rise and health awareness increases, selenium-enriched foods are offering multiple health benefits, including providing antioxidant stress relief, reducing the risk of cancer, maintaining cardiovascular health, delaying cognitive decline, supporting thyroid function, and enhancing the immune system [4,5,9,43]. This presents promising prospects for the development of selenium-fortified aquatic products. In recent years, the aquaculture sector has incorporated inorganic selenium, organic selenium (SeMet and Se yeast), and bio-nano selenium into feed formulations. These additions have significantly increased selenium levels in aquatic animals, promoting the green and sustainable development of selenium-enriched aquaculture (Table 2). However, dietary selenium supplementation in feed can improve growth performance, enhance antioxidant and immune capacity, maintain gut microbiota balance, and improve meat quality in farmed animals. Dietary selenium supplementation is positively correlated with overall animal health [44]. Selenium concentrations in the muscle tissue of *Platyrhinoidis triseriata* from the Eastern Pacific Ocean range from 0.14 to 1.31 μg/g [45]. In fish, the concentration of selenium in muscle tissue is influenced by dietary selenium content, selenium source, and phylogeny [46]. Different selenium sources exhibit variations in bioavailability. Consequently, selecting appropriate selenium sources is critical to enhancing selenium content in farmed animals. A comparative analysis of the selenium content of marine and freshwater fish. On average, the selenium content of marine fish muscle tissue was significantly higher than that of freshwater fish, at 0.412 ± 0.143 mg/kg and 0.215 ± 0.096 mg/kg, respectively [47]. In Rio Grande do Sul, the recommended daily selenium intake of 55 μg/d for adults can be achieved by consuming at least 134 g/d of freshwater fish or 82 g/d of marine fish filet [47]. Consumption of the analyzed selenium-enriched fish is safe for human health. According to the 2023 edition of China’s Dietary Reference Intakes for Nutrients [48], the estimated average requirement (EAR) for selenium in adults aged 18 is 50 μg/d, while the recommended nutrient intake (RNI) is 60 μg/d and the tolerable upper intake level (UL) is 400 μg/d. The accumulation of selenium in aquatic animals differs based on the concentrations of selenite, SeMet, SeNP, and Se-yeast in feed. The authors estimated the dietary intake of fish with corresponding adequate intake (AI) and UL (Table 2).

## 3. Benefits for Human Health

### 3.1. Function of Selenoproteins in Aquatic Animals

Selenium is a vital component in the production of selenoproteins. Most selenoproteins are biologically active antioxidant enzymes which protect the body from oxidative damage by neutralizing harmful reactive oxygen species and eliminating free radicals. These enzymes regulate redox homeostasis and are vital for maintaining cellular health. While it is well known that humans possess 25 selenoproteins, fish have been found to harbor 30–37 selenoproteins, representing one of the largest known protein repertoires [62], 24 selenoproteins share similarities with their human counterparts, and other selenoproteins are unique in fish (Table 3). They enter the body through dietary intake, participate in selenoprotein synthesis pathways, and ultimately play vital roles in maintaining health, such as scavenging peroxides, regulating thyroid hormones, mediating apoptosis, and enhancing male and female reproductive functions (Table 3).

A recent investigation into the selenoproteinome of fish revealed that supplementing *Oncorhynchus mykiss* with yeast selenium resulted in the detection of a total of 28 selenoprotein genes in muscle and liver tissues. The expression of the SELENOW gene was found to be highly correlated with muscle growth [63]. Additionally, fish-specific selenoprotein functions have been identified. Exploring the expression mechanisms of selenoprotein genes in grass carp revealed that SELENOL, SELENOU, and SELENOE play a prominent role in regulating lipid metabolism in muscle tissue following dietary selenium supplementation [64]. Research indicates that adding 3.5 mg/kg of Nano-Selenium to feed significantly increases the expression of GPX9 and SELENOE in the muscle of *Ictalurus punctatus*, demonstrating the role of selenoproteins in promoting muscle development and regulating oxidative stress [18]. Furthermore, fish oil contains high levels of unsaturated fatty acids such as EPA and DHA, which are highly susceptible to oxidation. Selenium promotes muscle antioxidant defense by forming selenoproteins that maintain cellular homeostasis, thereby preserving the quality and nutritional value of fish meat [65]. A healthy diet incorporating fish nutrients and selecting species with higher selenium content allows for more efficient intake of fish selenoproteins, given the significant variation in selenium levels among different species of fish.

**Table 3:** The connection between selenoproteins in aquatic animals and humans.

Selenoproteins	Abbreviation	Aquatic Animals	Benefits for Human Health
Glutathione peroxidase	GPX1	*Danio rerio*, *Sparus aurata* [66], *Ictalurus punctatus* [18]	Reduce cellular H_2_O_2_ and lipid peroxides [4,7]
	GPX2	*Oreochromis niloticus* [66], *Ictalurus punctatus* [18]	Reduce intestinal peroxides [4,7]
	GPX3	*Pelteobagrus fulvidraco* [66], *Ictalurus punctatus* [18]	Reduces plasma hydrogen peroxide [4,7], delaying aging [67], prevents rheumatoid arthritis [68]
	GPX4	*Gadus morhua*, *Thunnus maccoyi*, *Coho salmon* [66]	Preventing iron-induced ferroptosis [4,7]
	GPX6	*Oryzias melastigma* [69]	Enhance sperm capacitation and acrosome reaction [70]
Iodothyronine deiodinase	DIO1	*Ctenopharyngodon idella* [71,72]	Regulate thyroid hormone levels [73,74]
	DIO2	*Ictalurus punctatus* [18], *Ctenopharyngodon idella* [71]	Activate the biosynthesis of thyroid hormones in peripheral tissues [73,74]
	DIO3	*Ictalurus punctatus* [18], *Ctenopharyngodon Idella* [71]	Inhibit thyroid hormone production through selective deiodination. [73,74]
Thioredoxin reductase	TXNRD1	*Pelteobagrus fulvidraco* [66]	Reduce thioredoxin levels and regulate antioxidant activity [4,73]
	TXNRD2	*Pelteobagrus fulvidraco* [66,75]	Regulating transcription factors and modulating mitochondrial-mediated apoptosis mechanisms [7,73]
	TXNRD3	*Pelteobagrus fulvidraco* [75]	Regulating male fertility [76]
Selenophosphate synthetase 2	SEPHS2	*Danio rerio* [77]	Participates in the synthesis of all selenoproteins [73,74]
Methionine-R-sulfoxide reductase 1 (Selenoprotein R)	MSRB1 (SELENOR)	*Salmon fish* [66], *Pelteobagrus fulvidraco* [78]	Participates in antioxidant defense and regulates redox processes [73,74]
15 kDa Selenoprotein (Selenoprotein F)	15 kDa (SELENOF)	*Pelteobagrus fulvidraco* [66,78]	Associated with protein folding and secretion processes [79]
Selenoprotein H	SELENOH	*Ictalurus punctatus* [18], *Pelteobagrus fulvidraco* [78]	exhibits protective effects against oxidative stress, cellular senescence, and intestinal tumorigenesis [80]
Selenoprotein I	SELENOI	*Ictalurus punctatus* [18], *Pelteobagrus fulvidraco* [78]	Participates in liver phospholipid metabolism [81], control colitis and colorectal cancer [82]
Selenoprotein K	SELENOK	*Oreochromis niloticus* [66], *Ctenopharyngodon idella* [72]	Mediate neuromodulation and cognitive function, prevent Alzheimer’s disease (AD) [83]
Selenoprotein M	SELENOM	*Pelteobagrus fulvidraco* [66,78]	Regulating antioxidant stress and inflammation in pathological processes [84]
Selenoprotein N	SELENON	*Pelteobagrus fulvidraco* [78] *Danio rerio* [85]	Modulating endoplasmic reticulum stress to prevent muscle dysfunction [86]
Selenoprotein O	SELENOO	*Pelteobagrus fulvidraco* [78]	Increase neutrophil levels to alleviate liver inflammation [87]
Selenoprotein P	SELENOP	*Danio rerio* [66] *Ictalurus punctatus* [18], *Pelteobagrus fulvidraco* [75], *Magallana gigas* [88]	Delaying aging [67], reventing rheumatoid arthritis and osteoarthritis [68]
Selenoprotein S	SELENOS	*Pelteobagrus fulvidraco* [78]	Maintain ovarian function and improve female reproductive performance [89]
Selenoprotein T	SELENOT	*Pelteobagrus fulvidraco* [66] *Ictalurus punctatus* [18]	Maintain dopamine signaling in the brain [90]
Selenoprotein V	SELENOV	No	Body fat accumulation inhibitor, energy expenditure activator [91]
Selenoprotein W	SELENOW	*Oncorhynchus mykiss* [66], *Ctenopharyngodon idella* [72]	Regulate protein synthesis to prevent muscle atrophy [92]
Selenoprotein L	SELENOL	*Ctenopharyngodon idella* [64] *Oreochromis mossambicus* [93]	Selenoproteins not identified in humans, peculiar to aquatic animals.
Selenoprotein U	SELENOU	*Ctenopharyngodon idella* [64]	
Selenoprotein E	SELENOE	*Ictalurus punctatus* [18]	
Selenoprotein J	SELENOJ	*Oreochromis mossambicus* [93]	
Selenoprotein X	SELENOX	*Danio rerio* [94]	
Glutathione peroxidase	GPX7	*Bostrychus sinensis* [95]	
	GPX8	*Bostrychus sinensis* [95]	
	GPX9	*Ictalurus punctatus* [18]	

### 3.2. The Application of Aquatic Products in Human Health

**Figure 1:**
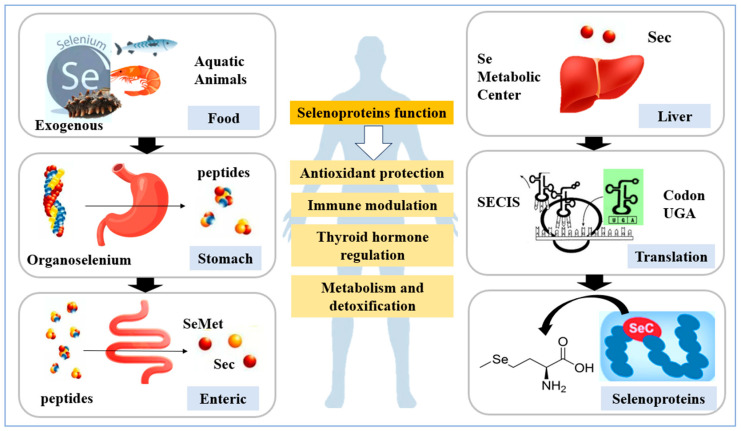
The relationship between dietary selenium and human selenoproteins.

Selenium is a vital trace element that is involved in numerous biological processes within the body. It serves as a crucial safeguard for maintaining normal physiological and biochemical functions. It regulates protein, lipid, and carbohydrate metabolism in animals by forming various selenoproteins, thereby ensuring animal health and enhancing immune function [96]. Appropriate selenium supplementation in feed enhances the production performance of aquatic animals and increases the nutritional value of selenium-enriched aquatic products, making them a preferred dietary source of trace elements. Analysis of the nutritional intake of 10,622 adults indicates that increased fish protein consumption improves the dietary quality of the US population [97]. Selenium-enriched oyster peptides, derived from selenium-rich oyster protein, contain 1.30–1.62 mg/kg of selenium. Consuming these bioactive peptides enhances the absorption of selenium. Additionally, selenium-enriched oyster peptides exhibit antioxidant properties and inhibit angiotensin-converting enzyme (ACE) activity, thereby lowering blood pressure [98,99]. Concerns regarding selenium status in the context of SARS-CoV-2 reveal that serum selenoprotein concentrations are significantly lower in patients with COVID-19 than in healthy adults. This suggests that selenium is not being transported efficiently to target organs, resulting in partial selenium deficiency in three major systems: the immune, endocrine, and central nervous systems [100]. As previously mentioned, SELENOP has been detected in the following species: *Danio rerio*, *Ictalurus punctatus*, *Pelteobagrus fulvidraco,* and *Magallana gigas*. The human body cannot synthesize selenoproteins independently and must obtain selenium from external sources in order to produce the selenoproteins it requires. Dietary supplementation with selenium-rich aquatic products, such as fish, helps to provide the necessary selenium for the body to synthesize the corresponding selenoproteins. After being digested and absorbed by the human body, selenium-enriched aquatic products (fish, shrimp, and sea cucumbers) are converted into selenocysteine. This substance is involved in synthesizing various selenoproteins in the body, and has antioxidant, anti-inflammatory, and regulatory effects on thyroid hormones and growth metabolism (Figure 1).

The Nutritional Prevention of Cancer Trial (NPCT), published by Clark and Combs in 1996, is one of the largest cancer prevention trials conducted on humans. Participants consumed 200 μg/day of selenium in the form of yeast tablets via dietary supplementation. Ultimately, the trial demonstrated a significant reduction in the incidence of colon, prostate, and lung cancers through this dietary intervention [101]. In 2022, primary liver cancer was the sixth most common cancer globally and the third leading cause of cancer death, with hepatocellular carcinoma (HCC) accounting for 75% to 85% of cases [102]. Sulfated polysaccharides (PS) extracted from sea cucumbers have been shown to inhibit HepG-2 cell proliferation by downregulating vascular endothelial growth factor (VEGF). They also induce apoptosis in hepatocellular carcinoma cells by downregulating B-cell lymphoma 2 (Bcl2) expression and upregulating Bcl2-associated X protein (BAX) and Bcl2 antagonist killer 1 (BAK) to induce apoptosis [103]. Harnessing the benefits of food as medicine, selenium-enriched sea cucumbers are a health food that delivers both nutrition and selenium efficiently. Additionally, seahorses (*Hippocampus*) are a valuable resource in Traditional Chinese Medicine (TCM), as they are believed to have medicinal properties that improve health by tonifying the kidneys, strengthening yang energy, and alleviating qi and blood deficiency [104]. As one of the most valuable Chinese medicinal materials, the development of selenium-enriched seahorses is a popular topic, as it is closely linked to human health. Like iodine, calcium, and zinc, selenium plays a crucial role in human health. Selenium-enriched aquatic products show promise as a safe and effective way to address selenium deficiency in the human diet.

## 4. Prospects for the Development and Utilization of Selenium-Enriched Aquatic Products

Against the backdrop of widespread health awareness, people no longer settle for mere sustenance but increasingly pursue a nutritious and wholesome diet. The growing emphasis on balanced nutrition and healthy lifestyles has led to a preference for selenium-rich foods, which enhance well-being and strengthen the foundation of physical and mental health. In the development trajectory of the global selenium market, demand for selenium-enriched foods is a pivotal driving force. This demand not only charts the market’s course but also directly propels upgrades to production and enhancements to supply capacity within the selenium-fortified food sector. Global Selenium Market Overview: The Selenium Market Size was estimated at 14.49 (USD Billion) in 2024. The Selenium Industry is expected to grow from 15.74 (USD Billion) in 2025 to 33.24 (USD Billion) by 2034. The Selenium Market CAGR (growth rate) is expected to be around 8.66% during the forecast period (2025–2034; available online: https://www.marketresearchfuture.com/reports/selenium-market-22837, accessed on 1 October 2025).

The selenium found in selenium-enriched aquatic products is predominantly organic selenium, primarily in the form of SeMet and Sec. These two forms can participate directly in human protein synthesis and are absorbed and utilized without requiring conversion, offering high bioavailability. Aquatic products are a good source of high-quality protein and omega-3 polyunsaturated fatty acids (EPA and DHA). When they are enriched with selenium, the nutritional benefits are enhanced. The Healthy China 2030 Plan explicitly advocates the development of nutrition-oriented agriculture in order to promote the production of premium specialty agricultural products. Selenium-enriched aquatic products are a prime example of functional nutritional agricultural products and have been incorporated into key agricultural industry upgrade projects across multiple regions [105]. Currently, selenium-enriched aquaculture is a well-established technical system. Through the use of selenium-fortified functional feed, the accumulation of selenium is gradually being achieved in a wider range of aquatic species, including Fish, shrimps, crabs, sea cucumbers, and abalone. In summary, the development and utilization of selenium-enriched aquatic products align with current consumer demands in terms of nutrition and health strategies, as well as the direction of the aquaculture industry in enhancing meat quality and functional benefits. This approach is further building high-value specialty aquatic brands and is set to become mainstream in the development of functional aquatic products.

## 5. Conclusions

Selenium is an essential trace element for the human body and is also closely associated with human nutritional balance, health maintenance, and disease prevention. Research indicates that supplementing animal feed with inorganic selenium, SeMet, se-yeast, and Nano-Se effectively increases the selenium content of farmed animals. Compared to inorganic selenium, organic selenium and Nano-Se demonstrate superior absorption and accumulation efficiency within animal tissues. Selenium-enriched aquatic products enhance selenium’s functional properties, building upon their inherent nutritional value and effectively supporting dietary selenium supplementation in humans. However, research on selenium-enriched aquatic products is limited in several ways. These include the variety of selenium sources, the wide range of dietary supplementation levels, the variations in animal breeds, and the differing accumulation rates of selenium across life stages. All of these factors contribute to significant variations in the selenium content of farmed animals. Furthermore, incomplete studies on selenium-containing proteins in aquatic products hinder public understanding of the nutritional value of selenium-enriched products.

Future research should optimize supplementation levels of different selenium sources further and delve into the molecular biological mechanisms of selenoproteins in various physiological processes. Linking aquatic products with the core physiological functions of selenoproteins enables consumers to clearly perceive the health benefits of dietary supplementation with selenium-enriched aquatic products, such as alleviating cardiovascular disease, enhancing antioxidant capacity, and boosting immunity. Secondly, there are significant variations in selenium absorption and metabolic capacity across different populations, which are closely linked to genetic polymorphisms associated with selenoprotein genes. In-depth research into the precise matching of genotyping with personalized selenium supplementation protocols is necessary to ensure the continued efficacy of supplementation. This will ultimately facilitate the development of more efficient and economical selenium-enriched products to address the global challenge of selenium deficiency.

## Figures and Tables

**Table 1:** Comparison of selenium content in aquatic products and other foods.

Food Types		Selenium Content Range (mg/kg)	References
Grains	Rice	0.177–0.205	[30]
	Bread	0.09–0.20	[31,32]
Fruits	Banana	0.043–0.057	[30]
	Apple	0.103 ± 0.001	[33]
Vegetables	Broccoli	0.061–0.118	[30]
	Turnip greens	0.061–0.073	[34]
Dairy products	Milk	0.107–0.162	[30]
	Cheese	0.070–0.0789	[35]
Meat	Pork	0.174–0.199	[36]
	Beef	0.27–0.67	[37]
Aquatic products	Tuna	1.6 ± 0.1	[38]
	Swordfish	1.4 ± 0.2	[38]
	Farmed salmon	1.03 ± 0.07	[38]
	Wild salmon	0.74 ± 0.08	[38]
	*Holothuria arguinensis*	4.26 ± 0.08	[39]
	*Holothuria forskali*	2.35 ± 0.13	[39]
	*Holothuria mammata*	3.22 ± 0.43	[39]
	Shrimp	0.126–1.741	[40]
	*Chlamys nobilis*	0.27–2.37	[41]
	*Aristichthys nobilis*	0.33	[42]
	*Cyprinus carpio*	0.27	[42]
	*Ctenopharyngodon idella*	0.19	[42]

**Table 2:** Selenium content in aquatic animals and recommended daily intake.

Animals	Weight (g)	Selenium in Feed	Selenium Concentration and Feeding Cycle	Selenium Content in Muscle and Body Wall (mg/kg)	References	AI (g/d)	UL (g/d)
*Ctenopharyngodon idella*	250.79 ± 1.57	Selenite, SeMet, Selenium nanoparticles (SeNP)	0.9 mg/kg, 60 days	0.395 ± 0.016, 0.592 ± 0.063, 0.629 ± 0.020	[14]	100–150	<635
*Ictalurus* *punctatus*	85.74 ± 5.75	Nanoselenium (Nano-Se)	5 mg/kg, 60 days	0.126 ± 0.02	[18]	476	<3175
*Scaphesthes macrolepis*	2.1 ± 0.03	Nano-Se	0.7, 1.47, 2.08, 60 days	0.19–0.20	[42]	300–316	<2000
*Nibea coibor*	11.34 ± 0.12	Sodium selenite	1.72 mg/kg, 8 weeks	0.25 ± 0.01	[49]	240	<1600
*Juvenile Catla catla*	6.41 ± 0.02	Nano-Se	1.08 mg/kg,	0.53 ± 0.02	[50]	114	<755
*Pelteobagrus fulvidraco*	7.55 ± 0.03	Sodium-selenite, Se-yeast, SeMet	0.25 mg/kg, 10 weeks	0.11 ± 0.01, 0.15 ± 0.02, 0.13 ± 0.01 (DW)	[51]	41–55	<270
	49.79 ± 0.65	Sodium selenite, Se-yeast Se-enriched *Spirulina platensis*	1.08, 1.14, 1.12 mg/kg, 50 days	0.91 ± 0.02, 1.49 ± 0.02, 1.15 ± 0.02	[52]		
*Cyprinus carpio*	9.94 ± 0.03	SeNP	0.5 mg/kg, 8 weeks	0.65 ± 0.06	[53]	66–93	<660
	9.69 ± 0.12	Sodium selenite, SeMet, Nano-Se	0.7 mg/kg, 8 weeks	0.81 ± 0.02, 0.91 ± 0.01, 0.89 ± 0.03	[54]		
*Apostichopus japonicus*	1.34 ± 0.18	Bio-fermenting Se, SeMet	5 mg/kg, 30 days	0.215 ± 0.02, 0.235 ± 0.03 (DW)	[55]	20–33	<130
	0.11 ± 0.01	Se-yeast	1 mg/kg, 45 days	3.1 ± 0.05	[56]		
	70 ± 10	κ-Selenocarrageenan	2 mg/kg, 30 days	1.87 ± 0.02	[57]		
*Penaeus vannamei*	1.55 ± 0.04	SeNP	0.38 mg/kg 8 weeks	0.97 ± 0.08	[58]	62	<620
*Haliotis discus hannai*	1.57 ± 0.01	Organic Se (Sel-Plex)	0.322, 0.427, 0.596 mg/kg, 60 days	0.459 ± 0.02, 0.53 ± 0.05, 0.851 ± 0.07	[59]	71–130	<705
*Rachycentron canadum*	13.65 ± 0.40	Se-yeast	1.5, 3 mg/kg, 8 weeks	0.486–1.217	[60]	125	<824
*Eriocheir sinensis*	4.5 ± 0.4	Nano-Se	0.1, 0.4, 0.8 mg/kg, 90 days	0.22 ± 0.02, 0.23 ± 0.02, 0.25 ± 0.01	[61]	160–182	<1600

## Data Availability

No new data were created or analyzed in this study.
